# Renal denervation in the setting of heart failure

**DOI:** 10.1007/s10741-025-10489-z

**Published:** 2025-01-29

**Authors:** Franziska Koppe-Schmeißer, Karl Fengler, Karl-Patrik Kresoja, Philipp Lurz, Karl-Philipp Rommel

**Affiliations:** 1 Department of Cardiology, University Medical Center of the Johannes Gutenberg University, Langenbeckstraße 1, 55131 Mainz, Germany; 2 Department of Cardiology, Heart Center at University of Leipzig and Leipzig Heart Institute, Leipzig, Germany

**Keywords:** Renal denervation, Heart failure, HFpEF, Sympathetic nervous system, Hemodynamics

## Abstract

Renal Denervation (RDN) has emerged over the last decade as a third pillar in the treatment of arterial hypertension, alongside pharmacotherapy and lifestyle modifications. Mechanistically, it reduces central sympathetic overactivation, a process also relevant to heart failure. In this mini-review, we summarize the development of RDN for heart failure, discuss the current evidence supporting its effects, and provide an outlook on future developments.

## Introduction

Heart failure (HF) is a clinical syndrome with various manifestations and a complex pathophysiology, divided into different phenotypes primarily according to left ventricular ejection fraction (LVEF) [1]. It is highly prevalent, affecting more than 64 million people worldwide [2]. Effective medical treatment has been established over the past few decades with Angiotensin-converting enzyme (ACE) or Angiotensin- receptor Neprilysin-inhibitors (ARNI), beta-blockers, mineralocorticoid receptor antagonists (MRA), sodium-glucose cotransporter 2 (SGLT2) inhibitors and soluble guanylate cyclase (sGC) stimulators. However, even with treatments HF-related morbidity and mortality is still high. In addition, these therapies almost exclusively benefit patients with HF and reduced ejection fraction (HFrEF) or at least HF with mildly reduced ejection fraction (HFmrEF), whereas HF with preserved ejection fraction (HFpEF) with its varying comorbidities, remains a clinical challenge [3]. Arterial hypertension accounts for one of the most common comorbidities in patients with HFpEF besides diabetes and aging. It leads to an altered hemodynamic status with arterial and ventricular stiffening, increased pulsatile left ventricular (LV) load and elevated LV filling pressures [4–6]. Chronic overactivation of the sympathetic nervous system (SNS) is known to be a relevant component in both HF as well as arterial hypertension [7–9]. Renal sympathetic denervation (RDN) was developed in the early 2000s to reduce sympathetic overactivation. Originally intended for heart failure patients, its use pivoted toward the treatment of arterial hypertension, for which the technology has since accumulated robust evidence of effectiveness [10, 11]. When RDN was first used for patients with uncontrolled hypertension receiving 5 or more antihypertensive drugs with office-measured systolic blood pressure (SBP) > 160 mmHg, the blood pressure reductions were in the range of 25–30 mmHg after 6 months, where adequate sham controls were missing [12, 13]. The sham-controlled SIMPLICITY-3-HTN-study revealed a reduction of 14 mmHg of SBP in the intervention group receiving RDN, however, not different from the 12 mmHg reduction the sham control group [14]. Recent RDN trials, using more rigorous designs, including to avoid medication changes, test for medication adherence and new device technologies have unequivocally demonstrated a blood pressure reduction by RDN between 9–11 mmHg office SB and 5–9 mmHg ambulatory systolic blood pressure [10, 15–17].

More recent data indicate that RDN might also improve other sympathetically mediated cardiometabolic conditions, including heart failure [16–18]. In this mini-review, we discuss the current state of knowledge for RDN in heart failure both with preserved and reduced ejection fraction and give an overview of future developments.

## Sympathetic nervous system in heart failure

The sympathetic nervous system (SNS) and the renin-angiotension-system (RAAS) are major components in the development of chronic heart failure, characterized by an imbalance to maintain cardiac output and appropriate organ perfusion [1]. There is an interplay between the SNS and RAAS, involving pathways between mechanosensitive baroreceptors, detecting changes in blood pressure, and chemoreceptors, detecting rising CO_2_ in the carotid arteries and peripherally in the kidneys. Afferent nerve signaling is integrated into hypothalamic and autonomic regulatory centers within the central nervous system. Consequently, efferent nerve activity via the kidney can promote vasoconstriction, salt and water retention, thus increasing cardiac chamber volumes, muscle mass and interstitial fibrosis [19, 20]. In the context of HF, both HFpEF and HFrEF, there is an inappropriate overactivation of the SNS from a variety of etiologies, resulting in cardiac hypertrophy, fibrosis, arrhythmias, tissue edema and vasoconstriction [21]. It is known, that in HF, activation of renal sympathetic efferent nerves causes renin release, sodium and water retention, thus leading to reduced renal blood flow with renal sympathetic activation causing high levels of angiotensin II (ATII)[19]. This in turn affects the central nervous system and results in elevation of global sympathetic tone [19]. The early stages of heart failure are characterized by reduced ability to increase natriuresis in response to salt load, thus leading to sodium retention [22, 23] via overactivation of the sodium reabsorption in the proximal tubule level is a key mechanisms of the sodium retention in HF, due to an imbalance of angiotensin II (AT II) and nitrite oxide (NO) and it is reversed by converting enzyme inhibitors (CEIs) reducing levels of AT II and increasing levels of NO [24, 25], which has vasorelaxant characteristics and is a physiological antagonist of AT II in the kidney [26]. According to recent studies, renal functional reserve (RFR), i.e., glomerular vasodilatory response to amino acid infusion, could serve as an index of the intrarenal balance between AT II and NO [25, 27], which is already impaired in early stages of HF [28] and can be restored and improved by CEIs. Hence, directly attenuating SNS overactivation appears to be a promising treatment strategy in HF.

## Renal sympathetic denervation in heart failure

RDN represents a one-time procedure destructing nerves surrounding the renal vasculature by using radiofrequency or ultrasound [29–31]. This leads to a reduction in renal afferent and efferent sympathetic nerve activity [21], potentially counteracting neurohormonal overactivation in HF [16, 32].

By denervation of efferent sympathetic fibers to the kidney, RDN ameliorates the sympathetic tone of the kidney itself, measured as reduced renal noradrenalin spillover. Moreover, evidence suggests that the more important effect of RDN is reducing the global sympathetic tone, via afferent effects and central modulation of autonomic tone, measured by muscle sympathetic nerve activity, improved baroreflex sensitivity, and heart rate variability [16, 32]. In addition, RDN has been suggested to exert cardioprotective effects via a reduction of neprilysin activity and natriuretic peptide degradation in rodent models [21, 33].

Studies establishing RDN as a therapy for hypertension [10] have also demonstrated beneficial effects on additional physiological endpoints relevant to heart failure patients. These include improved heart rate, insulin resistance, renal function and reduced microalbuminuria, and mitigation of conditions like sleep apnea and cardiac arrhythmias [34, 35].

## Renal sympathetic denervation in patients with HFrEF

**Fig. 1:**
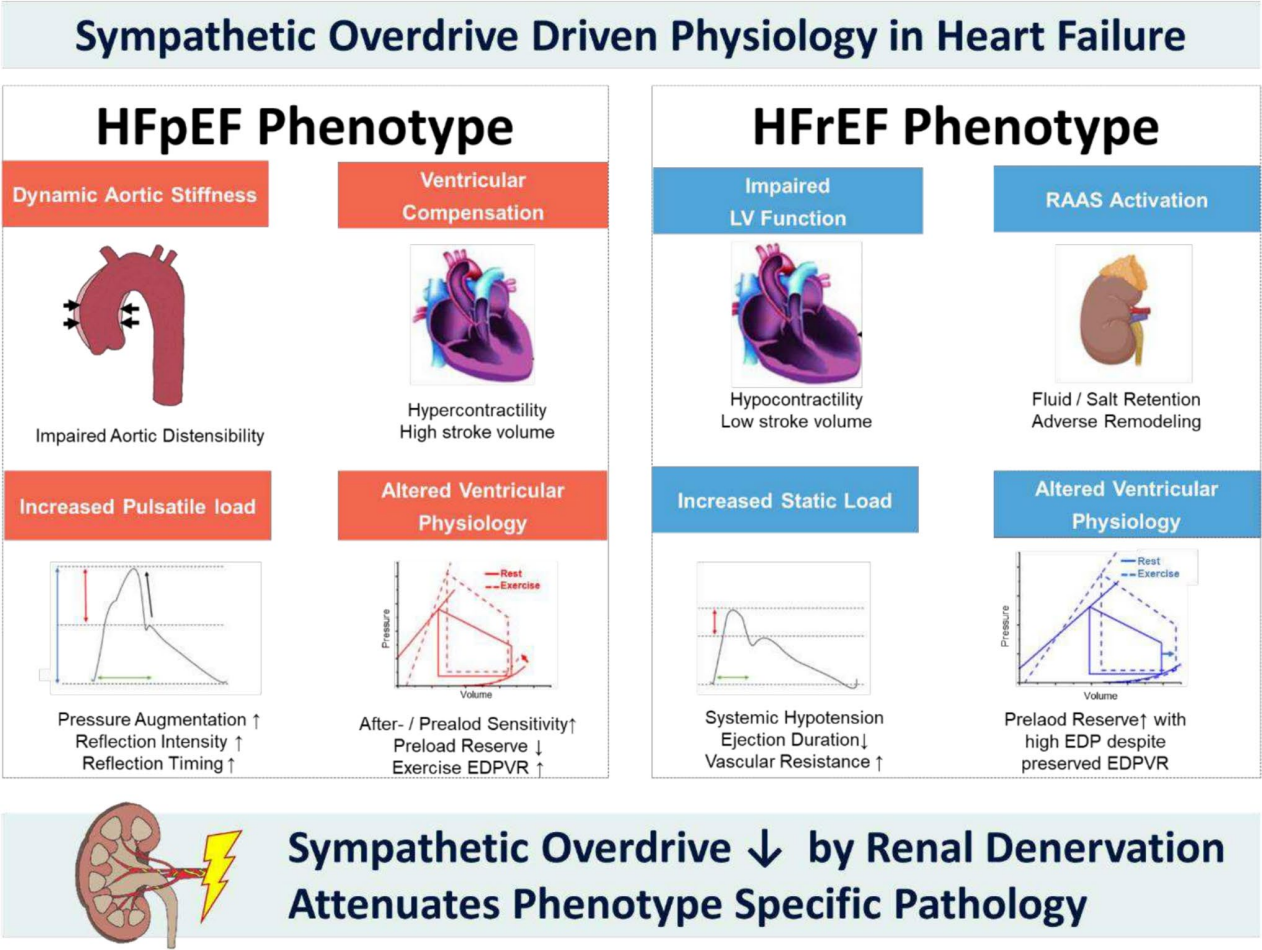
**Central Illustration** Phenotype specific pathologies in heart failure addressed by renal denervation. EDP = end-diastolic pressure; EDPVR = end-diastolic pressure–volume-relationship; HFpEF = heart failure with preserved ejection fraction; HFrEF = heart failure with reduced ejection fraction; LV = left ventricular; RAAS = renin–angiotensin–aldosterone system

Neurohormonal overactivation with increased levels of norepinephrine spillover and muscle sympathetic activity is well established as central pathophysiology in HFrEF (Fig. 1) [8, 36]. In fact, pharmacotherapies attenuate the downstream effects of SNS activation by blocking different aspects of the neurohormonal cascade. In contrast, RDN provides the potential to directly and permanently attenuate SNS independent of drug interactions and patient adherence [37]. Both, arterial and cardiopulmonary receptors normally restrain sympathetic outflow. Cardiopulmonary baroreflex control of sympathetic nerve activity is weakened in HF [38]. This may lead to reduced inhibitory influence of these receptors in HF and result in sympathetic excitation in HF [39]. It has been shown that RDN restores vagus nerve control over the heart by restraining the sympathetic activation of efferent nerves to the heart [19]. As such, the suggested reduction in RAAS and neprilysin activity with RDN very much mirror pharmacologic therapies established to improve outcomes in HFrEF.

A number of small sized studies, including six randomized trials have been published on RDN in patients with HFrEF. While inclusion criteria and study endpoints differed and findings were heterogeneous, a study-level meta-analysis found improvements in LV ejection fraction, 6-min walking distance, natriuretic peptide levels, NYHA class, heart rate with RDN. In addition, structural benefits, with reduction in dimension of LV, left atrium and LV wall thickness were suggested [40]. Interestingly, RDN in patients with HFrEF did not reduce systolic or diastolic blood pressure, suggesting an improvement in cardiac structure and function, independently of blood pressure reduction and without symptoms of hypotension in these patients [13, 40, 41]. Beneficial effects of RDN in HFrEF seem to be more prominent in early stages of HF [42]. However, RDN has also anecdotally been suggested to benefit more advanced phenotypes by reducing the burden of ventricular arrhythmias [43, 44].

All these studies share limitations, including small sample sizes, reliance on early RDN technologies, and the lack of a sham-controlled comparator arm and blinded outcome assessment. These latter elements are critical for reducing placebo and Hawthorne effects in RDN research and have become standard in randomized controlled trials for interventional devices. The RE-ADAPT-HF trial (NCT04947670) was designed as a prospective, multicenter, randomized, blinded, sham-controlled feasibility study of RDN in patients with HF, addressing many of these limitations. Unfortunately, the study has been terminated due to slow enrollment. Future studies are eagerly awaited to investigate a potential role for RDN in HFrEF.

## Renal sympathetic denervation in patients with HFpEF

HFpEF has been challenging to treat, with SGLT2 inhibitors only recently shown to reduce heart failure hospitalizations. However, residual morbidity and mortality remain high, hyper-polypharmacy is common and linked to adverse outcomes, and device therapies that directly target underlying pathomechanisms are well-positioned to address this unmet need [1, 3, 45].

Also, HFpEF is associated with elevated sympathetic tone, particularly when arterial hypertension is present [46, 47]. However, the failure of trials on pharmacological RAAS modulation to demonstrate robust clinical benefits may reflect the syndrome’s well-known heterogeneity, where various comorbidities and both central and peripheral pathologies lead to differing levels of neurohormonal activation. Alternatively, it suggests that SNS activation may have distinct pathological effects within specific HFpEF phenotypes [48, 49]. Indeed, recent findings highlight significant hemodynamic differences in HFpEF patients with LV ejection fractions above or below 60%. Patients with lower ejection fractions exhibit reduced LV contractility as well as impaired ventriculo-arterial coupling and increased myocardial fibrosis, with exercise adaptations resembling those in HFrEF; this group also shows greater responsiveness to RAAS inhibition in clinical trials [50]. By contrast, patients with higher LV ejection fractions display a hypercontractile state, excessive LV afterload, and limited preload reserve, primarily due to increased large artery stiffness, which raises pulsatile LV afterload, impairs LV filling, and promotes ventricular remodeling and stiffening [4–6, 51, 52]. Differences in patients with HFpEF and higher EF are related to smaller heart size, increased diastolic stiffness and leftward shift in EDPVR (End-Diastolic-Pressure–Volume-Relationship). This leads to increased sensitivity to preload and afterload with pronounced blood pressure swings [53]. Therapies such as neurohormonal antagonists that work through reverse remodelling are less effective in this group of patients [51, 54]. Observational data suggest that large artery stiffness in HFpEF has a dynamic, sympathetically mediated component, as RDN in patients with resistant hypertension has been shown to reduce aortic stiffness markers, improve diastolic LV properties, and reduce LV mass [21]. When comparing HFpEF patients with resistant hypertension to those without HF, HFpEF patients exhibited increased stroke volume, lower aortic distensibility, higher myocardial work indices, and greater blood pressure variability (an indicator of elevated sympathetic tone) [33]. While the blood pressure response to RDN was similar across groups, HFpEF patients experienced normalization of stroke volumes, improved aortic distensibility, better LV work profiles, and improvements in NYHA class and natriuretic peptide levels. Wave separation analysis demonstrated impaired total arterial compliance and aortic impedance, with an unfavorable pulsatile LV load in HFpEF patients, which was partially normalized after RDN [55]. Interestingly, the reduction in BP after RDN may partly rely on a reduction in stroke volume [56].

These findings collectively suggest that, unlike in HFrEF where RAAS activation plays a larger role, SNS overactivation in HFpEF predominantly impacts LV-arterial interaction, particularly in patients with higher LV ejection fractions (Fig. 1). Thus, RDN may be especially effective in attenuating the central hemodynamic alterations in this subgroup [57].

Additionally, SNS attenuation through RDN could benefit HFpEF patients by enhancing splanchnic and venous blood pooling or by reducing atrial fibrillation burden [58].

Moreover, experimental studies have also linked sympathetic nervous system (SNS) overactivation in the kidneys to altered glucose metabolism, driven by increased tissue glucose uptake mediated by SGLT2 upregulation, which can be reversed by RDN [59, 60].

This is particularly relevant given that pharmacological SGLT2 inhibition has emerged as a powerful strategy in HF management. Whether RDN and pharmacological SGLT2 inhibition exert synergistic effects in human HF remains an intriguing area for future research.

To date, only one dedicated RCT has evaluated the effects of RDN in HFpEF. This single-center study enrolled 25 patients, with 18 undergoing RDN using a first-generation device. The unblinded study was terminated early due to recruitment challenges and was underpowered to detect meaningful improvements in the primary endpoint, quality of life at 12 months. Nonetheless, a greater proportion of patients in the RDN group showed improved exercise capacity and diastolic function at 3 months [61].

The ongoing UNLOAD-HFpEF trial (NCT05030987) is a multicenter, randomized, sham-controlled, triple-blinded study designed to assess the hemodynamic and clinical effects of RDN in 68 patients with HFpEF. The primary endpoint is exercise-induced pulmonary capillary wedge pressure at 20 W during dynamic exercise testing. Secondary endpoints include MRI findings, invasive pressure–volume analyses, and pulmonary artery pressure sensor data, which aim to enhance our understanding of the role of RDN in HFpEF.

### RCTs: HFrEF + RDN

**Table:** 

Study	Year	Country	LVEF	NYHA	Number of patients	Follow-up time	Treatment	Findings
Chen [62]	2017	China	< 40%	II-IV	60	6	RDN + OMT/OMT	LVEF 41.9 ± 7.9% (RDN) vs. 31.2 ± 5.5% (control), *p* < 0.001 SMWD (*p* = 0.043), NYHA class (*p* < 0.001), NT-proBNP (*p* < 0.001) and office heart rate (*p* = 0.008) sign. reduced in RDN vs. control group
Spadaro [63]	2019	Brazil	< 40%	II-III	17	9	RDN + OMT/OMT	Composite of all-cause death, myocardial infarction, stroke, need for renal artery invasive treatment, or worsening renal function occured in 36.4% in RDN group vs. 50.0% (control) (*p* = 0.6) at 9 months, clinical, laboratory, functional, echocardiographic, and quality of life parameters were similar between groups
Gao [64]	2019	China	< 40%	II-III	60	6	RDN + OMT/OMT	LVEF increased from 36.0 ± 4.1% to 43.8 ± 7.9% (RDN) (*p* = 0.003), SMWD increased from 152.9 ± 38.0 m before RDN to 334.3 ± 94.4 m after RDN (*p* < 0.001), systolic BP (RDN) decreased from 138.6 ± 22.1 mmHg to 123.2 ± 10.5 mmHg (*p* = 0.026) and diastolic BP from 81.1 ± 11.3 mmHg to 72.9 ± 7.5 mmHg (*p* = 0.032)
Drozdz [65]	2019	Poland	< = 35%	II-IV	20	12	RDN + OMT + CRT/ OMT + CRT	no significant differences in LVEF, BP, SMWD and NT-proBNP concentration 6 and 12 months after RD or control
Feyz[66]	2021	Netherlands	< = 35%	II-IV	49	6	RDN + OMT/OMT	combined endpoint of cardiovascular death, rehospitalisation for heart failure, and acute kidney injury in 8.3% (RDN) vs 8.0% (OMT) (*p* = 0.97), no significant changes in cardiac sympathetic nerve activity as measured using ^123^I-MIBG
Pietila- Effati [67]	2022	Finland	< 45%	III-IV	10	6	RDN + OMT + CRT/OMT + CRT	RDN did not show benefit for patients with severe heart failure (NYHA III and IV) who were non-responders to CRT
Mahfoud (RE-ADAPT-HF)	2021	Germany	< 45%	II-III	144	12	RDN + OMT/OMT	Study terminated due to slow enrollment

Adapted from Li et al [40]

*LVEF* Left ventricular ejection fraction, *SMWD* 6-min walking distance, ^*123*^*I-MIBG* iodine-123 meta-iodobenzylguanidine

### RCTs HFpEF + RDN

**Table:** 

Study	Year	Country	LVEF	NYHA	Number of patients	Follow-up time	Treatment	Findings
Patel[61] (RDT-PEF)	2016	London	> 50%	II-III	25	12	RDN + OMT/OMT (unblinded)	Study was terminated early because of difficulties in recruitment and was underpowered to detect whether RD improved the endpoints of quality of life, exercise function, biomarkers, and left heart remodelling Patients in the RDN group showed improved exercise capacity and diastolic function at 3 months
Lurz (UNLOAD-HFpEF)	2024	Germany	> 55%	II-III	68	24	RDN + OMT/OMT (sham-controlled, blinded)	primary endpoint is exercise-induced pulmonary capillary wedge pressure at 20 W during dynamic exercise testing. Secondary endpoints include MRI findings, invasive pressure–volume analyses, and pulmonary artery pressure sensor data

## Conclusion

In summary, RDN shows promise as an adjunctive therapy for both HFrEF and HFpEF, as it directly attenuates SNS overactivation, addressing phenotype-specific pathomechanisms. An ongoing RCT in HFpEF aims to enhance our understanding of RDN’s effects and its potential clinical utility in HF management. A properly sized, dedicated trial of RDN in HFrEF is warranted.

## Data Availability

No datasets were generated or analysed during the current study.
